# *ICOS *gene polymorphisms are associated with sporadic breast cancer: a case-control study

**DOI:** 10.1186/1471-2407-11-392

**Published:** 2011-09-15

**Authors:** Fengyan Xu, Dalin Li, Qiujin Zhang, Zhenkun Fu, Jie Zhang, Weiguang Yuan, Shuang Chen, Da Pang, Dianjun Li

**Affiliations:** 1 Department of Immunology, Harbin Medical University, Harbin 150081, China; 2 Institute of Cancer Prevention and Treatment, Harbin Medical University, Harbin 150081, China; 3 Department of Surgery, the Third Affiliated Hospital of Harbin Medical University, Harbin, 150081, China

## Abstract

**Background:**

*Inducible costimulator *(*ICOS*), a costimulatory molecular of the *CD28 *family, provides positive signal to enhance T cell proliferation. Its abnormal expression can disturb the immune response and entail an increased risk of cancer. To investigate whether single nucleotide polymorphisms (SNPs) in the *ICOS *gene are associated with sporadic breast cancer susceptibility and progression in Chinese women, a case-control study was conducted.

**Methods:**

In the study cohort, we genotyped five SNPs (rs11889031, rs10932029, rs4675374, rs10183087 and rs10932037) in *ICOS *gene among 609 breast cancer patients and 665 age-matched healthy controls. Furthermore, the positive results were replicated in an independent validation cohort of 619 patients and 682 age-matched healthy controls. Polymerase chain reaction-restriction fragment length polymorphism (PCR-RFLP) was used to determine the genotypes.

**Results:**

In rs10932029, compared with TT genotype and T allele, the CT genotype and C allele showed a significantly increased risk of breast cancer (P = 0.030, OR = 1.467, 95% CI 1.037-2.077; P = 0.017, OR = 1.481, 95% CI 1.070-2.049, respectively), and the associations were also significant in the validation cohort (P = 0.002, OR = 1.693, 95% CI 1.211-2.357; P = 0.003, OR = 1.607, 95% CI 1.171-2.204, respectively). Haplotype analysis showed that CTCAC haplotype containing rs10932029 T allele had a lower frequency in cases than in controls (P = 0.015), whereas haplotype CCCAC containing rs10932029 C allele was more common in cases than in controls (P = 0.013). In the analysis of clinicopathologic features, rs11889031 CT genotype and T allele were associated with progesterone receptor (PR) status and lymph node metastasis, which were further supported by our validation cohort. Moreover, some haplotypes were associated with estrogen receptor (ER) and PR statuses.

**Conclusions:**

These results indicate that *ICOS *gene polymorphisms may affect the risk of breast cancer and show that some SNPs are associated with breast cancer characteristics in a northern Chinese population.

## Background

Breast cancer is one of the most common malignant tumors contributing to the high mortality of females worldwide. The etiology of breast cancer is a complex combination of both environmental and genetic factors, so the determination of genetic polymorphism provided a new way to investigate the etiology of such complex genetic disease. Accordingly, significant associations have been demonstrated on some gene polymorphisms with breast cancer risk [1]. So far, accumulating evidence convincingly emphasizes that the host immune system is involved in the regulation of cancer development and progression. T lymphocyte, whose function is central to the adaptive immune response, plays a critical role in immune surveillance of cancer cells [2,3]. Therefore, the molecules, in particular the costimulatory ones, mediating regulation of T-cell activity may influence cancer susceptibility [4]. *Inducible costimulator *(*ICOS*) molecule is a member of the *CD28 *family that generates indispensable secondary signals to determine the activation and development of the immune response.

The gene encoding *ICOS *is located on chromosome 2q33, which contains *CD28 *and *CTLA-4*, another two numbers of *CD28 *family. Not expressed by naive Th cells, *ICOS *is induced following T cell activation [5]. Interaction between *ICOS *and its ligand (*ICOS-L*; *CD275*), a molecule highly expressed on B cells and dendritic cells, provides costimulatory signal to induce T cell proliferation, secretion of various cytokines and up-regulation of cell surface molecules [6]. Blocking of *ICOS *with Abs or an ICOS-Ig fusion protein results in the inhibition of immune responses mediated by Th1 and Th2 [7,8]. Furthermore, a recent research revealed that impaired function of CD4^+ ^and CD8^+ ^T cells were observed in *ICOS*-deficient patients [9].

Although the polymorphisms in *ICOS *gene have been extensively studied in various diseases, including cancers [10,11], the association between *ICOS *gene polymorphisms and the risk of breast cancer remains unclear. To determine the key roles of *ICOS *in tumor immunity, we genotyped five potentially functional SNPs, including rs11889031, rs10932029 (IVS1+173), rs4675374, rs10183087 (c.602) and 10932037 (c.1624), and investigate their associations with both the risk and clinicopathologic features of breast cancer in Chinese women from Heilongjiang Province, northeast of China.

## Methods

### Study population

This study cohort contained 609 sporadic breast cancer cases and 665 healthy controls. All cases (mean age at 49.5 ± 10.1 years) with histopathologically confirmed breast cancer were recruited from the Department of Breast Surgery in the Third Affiliated Hospital of Harbin Medical University, and their pathological and clinical information were obtained from medical files (Table 1), the controls were frequency-matched to cases by age (mean age at 48.0 ± 9.9 years) and were volunteers without any history of cancer. Both breast cancer cases and healthy controls were hereditarily unrelated and were recruited from Heilongjiang Province, northeast of China. The validation cohort consisted 619 breast cancer cases (mean age at 49.9 ± 10.2 years) and 682 age-matched healthy controls (mean age at 48.9 ± 10.0 years), and the selection criteria for cases and controls was as described above. At recruitment, each subject had signed the written informed consent, and provided 5 ml venous blood from 2006 to 2010. The study was approved by the institutional ethical review board.

**Table 1:** Clinicalpathologic features of breast cancer patients

Feature	Study cohort	Validation cohort
	
	Case no (%)	Case no (%)
Tumor type		
IDC	523(85.88)	528(85.30)
MC	7(1.15)	6(0.97)
Intraductal carcinoma	40(6.57)	45(7.27)
Mucinous adenocarcinoma	14(2.30)	13(2.10)
Others	25(4.11)	27(4.36)
Tumor size(cm)		
TZ ≤ 2	210(34.48)	200(32.31)
2 < TZ ≤ 5	277(45.48)	289(46.69)
TZ > 5	29(4.76)	37(5.98)
Unknown	93(15.27)	93(15.02)
LN involvement		
Positive	263(43.19)	278(44.91)
Negative	334(54.84)	333(53.80)
Unknown	12(1.97)	8(1.29)
ER		
Positive	313(51.40)	361(58.32)
Negative	208(34.15)	207(33.44)
Unknown	88(14.45)	51(8.24)
PR		
Positive	382(62.73)	345(55.74)
Negative	134(22.00)	223(36.03)
Unknown	93(15.27)	51(8.24)
P53		
Positive	153(25.12)	144(23.26)
Negative	353(57.96)	420(67.85)
Unknown	103(16.91)	55(8.89)
C-erbB-2		
Positive	198(32.51)	187(30.21)
Negative	317(52.05)	379(61.23)
Unknown	94(15.44)	53(8.56)

Abbreviations: IDC = infiltrative ductal carcinoma; MC = medullary carcinoma; LN = lymph node; TZ = tumor size; ER = estrogen receptor; PR = progesterone receptor

### DNA extraction and genotyping

Genomic DNA was extracted from frozen whole blood using the universal genomic DNA Extraction Kit VER.3.0 (TaKaRa, Japan). Genotyping was performed by polymerase chain reaction-restriction fragment length polymorphism (PCR-RFLP) method. The polymorphic region was amplified by PCR using a T-Gradient Thermoblock PCR System (Biometra, Germany) in a 25 ul reaction solution containing 0.3 ug genomic DNA, 2.5 ul 10× PCR buffer (Mg2+ plus), 0.2 ul dNTPs mixture, 2.5 U TaqDNA polymerase (TaKaRa, Japan) and 0.1 ul of each primer (Invitrogen, China). Primers sequences of each SNP were rs11889031 (F:5'-CAAACTGAGAAGCGAGAG-3', R:5'-ATAAGTTCTAGAGCTCAGGG-3'), rs10932029 (F:5'-CCTCTGGTATTTCTTTCTCTTC-3', R:5'-ACAGGTAACTCCAAGCAGG-3'), rs4675374 (F:5'-TGTTTCATCTTGTGCTGG-3', R: 5'-ACCATGCAGTTACCTTCC-3'), rs10183087 (F:5'-TATGAAAGGCAATGGAGAGG-3', R:5'-ATGATAGTGAAATGCGGACAG-3'), rs10932037 (F:5'-CATTATCTATGTTTTCATGGTATT-3', R:5'-AGGCTATCTTGAAGGGCCAG-3'). Annealing temperature were rs11889031 (58°C), rs10932029 (55.8°C), rs4675374 (56°C), rs10183087 (58°C) and rs10932037 (61°C). The lengths of PCR products were 586 bp (rs11889031), 317 bp (rs10932029), 301 bp (rs4675374), 520 bp (rs10183087) and 445 bp (rs10932037). The PCR products were digested with restriction enzymes (NEB, UK) according to the manufacturer's instruction and analyzed by 2% (rs11889031, rs10932029, rs4675374, rs10183087) and 4% (rs10932037) agarose gel electrophoresis (Additional file 1, Figure S1). Restriction enzymes of each SNP were XmnI (rs11889031), Ddel (rs10932029), Ddel (rs4675374), Hpy188III (rs10183087) and NlaIII (rs10932037). The digested fragments length of each SNP were rs11889031 (T: 401+185 bp, C: 586 bp), rs10932029 (C: 220+97 bp, T: 317 bp), rs4675374 (C:252+49 bp, T:301 bp), rs10183087 (C:283+133+104 bp, A:283+237 bp) and rs10932037 (C: 270+86+70+19 bp, T: 270+156+19 bp). In order to confirm the accuracy of genotyping results, 10% of the samples of each SNP were randomly selected to be tested twice by different persons. Furthermore, 3% random samples of each SNP were confirmed by direct sequencing, and the reproducibility of both was 100%. SNPs with suggestive statistical significance in the study cohort were replicated in an independent validation cohort to validate the results.

### Statistical analysis

Genotype frequencies of five SNPs were tested for Hardy-Weinberg equilibrium (HWE). We used Haploview 4.1 to tag all common haplotypes and their frequencies in cases and controls. Associations between SNPs and breast cancer risk were estimated by odds ratio (OR) and 95% confidence interval (CI) using unconditional logistic regression with adjustment for age. Disease characteristics were compared in patient using the chi-square test and Fisher's exact test. Homozygotes for the major allele were the reference group, and then heterozygotes and minor allele homozygotes were compared with the reference group, respectively. All statistical tests were two-sided, and statistical significance was set at P < 0.05. Statistical analyses were performed using SPSS 16.0 software.

## Results

### *ICOS *gene polymorphisms and the risk of breast cancer

The genotype and allele frequencies of five SNPs in the study cohort are shown in Table 2. No deviation from HWE was found in the genotype distribution of the five SNPs in controls (P > 0.05). In rs10932029, compared with TT genotype and T allele, the frequencies of CT genotype and C allele were higher in cases than those in controls (P = 0.030, OR = 1.467, 95% CI 1.037-2.077; P = 0.017, OR = 1.481, 95% CI 1.070-2.049, respectively). However, no significant differences were found between the alleles and genotypes of the other SNPs and the risk of breast cancer. To confirm this genetic association, we replicated rs10932029 in an independent validation cohort, and the associations remained suggestive in the validation cohort (P = 0.002, OR = 1.693, 95% CI 1.211-2.357; P = 0.003, OR = 1.607, 95% CI 1.171-2.204, respectively). We further analyzed the association between haplotypes and the risk of breast cancer. The haplotypes with frequency > 1% are shown in Table 3. CTCAC (11889031 C, rs10932029 T, 4675374 C, 10183087 A, 10932037 C) was the most frequent haplotype appeared in cases and controls (35.7%), and the frequency of this haplotype was lower in patients than in healthy controls (P = 0.015). Moreover, CCCAC haplotype had a higher frequency in cases (P = 0.013).

**Table 2:** Genotype and allele frequencies of *ICOS *polymorphisms and their associations with breast cancer risk

SNP	Study cohort					Validation cohort			
	
	Genotype and allele	Cases n = 609(%)	Controls n = 665(%)	OR (95% CI)^5^	P value	Cases n = 619(%)	Controls n = 682(%)	OR (95% CI)^5^	P value
rs11889031^1^	CC	248(40.92)	281(43.03)	reference					
	CT	286(47.19)	293(44.87)	1.104(0.872,1.398)	0.411				
	TT	72(11.88)	79(12.10)	1.025(0.713,1.472)	0.896				
	C	782(64.52)	855(65.47)	reference					
	T	430(35.48)	451(34.53)	1.039(0.882,1.224)	0.647				
rs10932029	TT	523(85.88)	599(90.08)	reference		522(84.33)	614(90.03)	reference	
	CT	82(13.46)	64(9.62)	1.475(1.042,2.088)	0.029	95(15.35)	66(9.68)	1.693(1.201,2.350)	0.002
	CC	4(0.66)	2(0.30)	2.348(0.427,12.904)	0.326	2(0.32)	2(0.29)	1.171(0.164,8.353)	0.875
	T	1128(92.61)	1262(94.89)	reference		1139(92.00)	1294(94.87)	reference	
	C	90(7.39)	68(5.11)	1.490(1.076,2.063)	0.016	99(8.00)	66(5.13)	1.595(1.162,2.189)	0.004
rs4675374^2^	CC	136(22.48)	165(25.82)	reference					
	CT	320(52.89)	325(50.86)	1.193(0.907,1.571)	0.207				
	TT	149(24.63)	149(23.32)	1.216(0.882,1.677)	0.233				
	C	592(48.93)	655(51.25)	reference					
	T	618(51.07)	623(48.75)	1.099(0.939,1.286)	0.241				
rs10183087^3^	AA	438 (72.16)	487(74.01)	reference					
	AC	154(25.37)	155(23.57)	0.909(0.702,1.177)	0.469				
	CC	15(2.47)	16(2.43)	0.951(0.454,1.993)	0.894				
	A	1030(84.84)	1129(85.79)	reference					
	C	184(15.16)	187(14.21)	0.998(0.826,1.158)	0.795				
rs10932037^4^	CC	568(93.42)	618(94.93)	reference					
	CT	40(6.58)	33(5.07)	1.326(0.824,2.133)	0.245				
	TT	0	0	--	--				
	C	1176(96.71)	1269(97.47)	reference					
	T	40(3.29)	33(2.53)	1.315(0.823,2.100)	0.252				

^1 ^cases n = 606, missing n = 3; controls n = 653, missing n = 12.

^2 ^cases n = 605, missing n = 4; controls n = 639, missing n = 26.

^3 ^cases n = 607, missing n = 2; controls n = 658, missing n = 7.

^4^cases n = 608, missing n = 1; controls n = 651, missing n = 14.

^5^ORs were adjusted for age

Abbreviations: OR = odds ratio; CI = confidence interval

**Table 3:** *ICOS *haplotype (rs11889031, rs10932029, rs4675374, rs10183087, rs10932037) frequencies in cases and controls

*ICOS *haplotype					Frequency	Cases (n = 609)	controls (n = 665)	P value
				
rs11889031	rs10932029	rs4675374	rs10183087	rs10932037				
C	T	C	A	C	0.357	0.333	0.379	0.015
T	T	T	A	C	0.289	0.294	0.285	0.651
C	T	T	A	C	0.145	0.152	0.139	0.385
C	T	C	C	C	0.051	0.047	0.055	0.382
C	C	C	A	C	0.037	0.047	0.028	0.013
T	T	T	C	C	0.028	0.031	0.026	0.501
C	T	T	C	C	0.020	0.020	0.020	0.974
C	C	C	C	C	0.017	0.018	0.016	0.674
T	T	C	A	C	0.016	0.016	0.016	0.966
C	T	C	C	T	0.010	0.013	0.007	0.150

Abbreviations: OR = odds ratio; CI = confidence interval

### *ICOS *gene polymorphisms and clinicopathologic features

*ICOS *gene polymorphisms were also analyzed to establish their associations with clinicopathologic features, including lymph node metastasis, tumor size and the statuses of estrogen receptor (ER), progesterone receptor (PR), C-erbB-2 and P53. The positive results in our study cohort are shown in Table S1. In rs10932029, compared with TT genotype, the frequency of CT genotype was lower in C-erbB-2 positive cases than negative ones (P = 0.046), but this result failed to be confirmed in the validation cohort (Additional file 2, Table S1). In rs11889031, compared with CC genotype and C allele, CT genotype and T allele had lower frequencies in PR positive cases (P = 0.002; P = 0.017, respectively), furthermore, CT genotype and T allele had lower frequencies in lymph node involvement (P = 0.011; P = 0.038, respectively). And these positive results were also confirmed in our validation cohort (Additional file 2, Table S1). However, no statistical association was obtained between five SNPs and the tumor size, ER or P53 statuses.

We also analyzed the association between haplotypes and clinical features of cases. The frequency of CTCCC haplotype was higher in ER positive cases (P = 0.008) and the CTTAC haplotype had a lower frequency in both ER positive and PR positive cases (P = 0.034; P = 0.005, respectively). However, no significant association was observed between haplotypes and lymph node involvement, tumor size, C-erbB-2 or P53 statuses.

## Discussion

The etiology of breast cancer is complicated, in which the genetic factor plays an important role. Although high-penetrant susceptibility genes such as *BRCA1 *and *BRCA2 *demonstrate potent associations with the familiar breast cancer, many low-penetrant susceptibility genes predisposing to breast cancer remain to be elucidated. Previous studies showed that SNPs of *Cytotoxic T lymphocyte antigen -4 *(*CTLA-4*) and *B and T lymphocyte attenuator *(*BTLA*) might confer susceptibility to breast cancer, which suggests the critical role of costimulatory molecules in the development of breast cancer [12,13]. In addition, *CTLA-4 *and *ICOS *are located within a stretch of 100 kb on chromosome 2q33. Treated with CTLA-4 blockade, ICOS expressed higher on CD4^+ ^T cells from peripheral blood and tumor tissues of bladder cancer patients and this CD4^+^ICOS^hi ^T cell population produced higher IFN-γ, indicating that ICOS interacts with CTLA-4 and plays an important role in tumor immunity [14,15]. Thus, we predicted that *ICOS *gene polymorphisms might also be related to the risk of breast cancer. In this study, we analyzed five potentially functional SNPs in *ICOS *gene, including rs11889031C/T in promotor, rs10932029 C/T and rs4675374C/T in the intron1, rs10183087A/C and rs10932037 C/T in the 3'-untranslating region (UTR), and determine their associations with breast cancer. Here, we first report that some of the alleles, genotypes and haplotypes of the above SNPs are associated with the risk and clinicopathological features of breast cancer.

This case-control study on the variants in the *ICOS *gene revealed that only rs10932029 may confer susceptibility to breast cancer. The association between rs10932029 C/T and breast cancer risk found in the study cohort was also observed in the validation cohort. Patients carrying rs10932029 CT genotype and C allele had an increased risk of breast cancer, which indicates that CT genotype and C allele may play risk roles in breast cancer. Rs10932029 C/T and rs4675374 C/T are both located in intron1 of *ICOS*. Introns are important for mRNA processing and transporting. Many studies have demonstrated the presence of regulatory elements and splicing control elements in mammalian introns, particularly in the first intron. Moreover, mutations occurring in introns can induce the aberrant splicing due to the disruption of splicing enhancers and alteration of the pre-mRNA, as a result, impair the translation efficiency [16-18]. Rs10932029 is a well studied variant in some diseases. Interestingly, one study demonstrated that this SNP could influence the expression of *CTLA4 *isoforms [19], but later study could not confirm this conclusion [20]. Although a correlation between rs10932029 and breast cancer risk was ascertained in our study, the mechanism underlying this result is not immediately evident. The potential explanation might be concluded as follows. Firstly, this polymorphism might regulate *ICOS *mRNA processing and translation. Secondly, CT genotype and C allele might down-regulate the ICOS expression and then increase the breast cancer risk. However, rs4675374 located in the same intron did not show any association with the risk of breast cancer.

Allelic variants located in the promoter region may change the motif of functional DNA binding sites and thereby affect their affinities for the relevant transcription factors. Notably, with a location in the promoter region, rs11889031 is situated in the NF-κB binding site [21]. However, although this potentially functional variant may affect the function of the gene, our data did not show any association between this SNP and the risk of breast cancer.

Rs10183087 A/C and rs10932037 C/T are both located in the 3'-UTR of the *ICOS*. Multiple regulatory elements in 3'-UTR can affect mRNA stability and degradation as well as nuclear export [22]. Using the PupaSuite software [23], rs10183087 is speculated to be located in the exonic splicing enhancers(ESE), so it may influence mRNA splicing, eventually, affect protein function. One previous study showed that rs10183087 was associated with delayed graft function [24], but no relation was found in malignant melanoma susceptibility [11]. Our study also did not show any statistical significant association between this SNP and the risk of breast cancer. Therefore, whether this SNP affect *ICOS *function needs to be further studied. Additionally, rs10932037A/C was located in a MicroRNA-binding site. Altering the strength of miRNA binding, SNPs in 3'UTR targeted by miRNAs were associated with the risk of breast cancer [25,26]. In line with this hypothesis, one study had verified that rs10932037 can regulate the expression of *ICOS *mRNA [19]. However, we failed to find the association between rs10932037 and the susceptibility to breast cancer. The correlation between rs10932037 and genetic susceptibility to a variety of diseases has been widely studied, but the results were inconsistent. Rs10932037 was proposed to be associated with kidney graft survival and the outcome of hematopoietic stem cell transplantation (HSCT) [24,27], but another study about gastric mucosa-associated lymphoid tissue lymphoma did not show any significant association [10]. Considering the minor allele frequency of rs10932037 was 2.53% in controls, further study using a large sample size is needed to confirm this association.

We further analyzed the association between haplotypes and the risk of breast cancer. We found that CTCAC haplotype containing rs10932029 T allele appeared to be protective against breast cancer, whereas CCCAC haplotype containing rs10932029 C allele may be a risk factor in breast cancer. This result also highlighted the important role for rs10932029.

In addition, we found that ICOS gene polymorphisms were associated with clinicopathologic features of breast cancer patients. For rs10932029, a correlation was found between it and C-erbB-2 status in the study cohort, but no relation was found in the validation cohort. As it is known, the over-expression of C-erbB-2 can lead to insensitive endocrine treatment, the recurrence and metastasis of tumor, resulting in poor prognosis and overall survival [28,29]. Nevertheless, in consideration of the converse results in two independent cohorts, whether rs10932029 was associated with C-erbB-2 status needs to be further investigated. We also found that rs11889031 was associated with PR status and lymph node metastasis in both study and validation cohorts. The expression of ER or PR has long been regarded as predictive markers of breast cancer endocrine therapy [30,31]. Moreover, cases of positive lymph node metastasis have higher mortality [32]. So our data indicate that genotypes and alleles in rs11889031 may be important in forecasting the prognosis of breast cancer.

In the analysis of the association between haplotypes and clinicopathlogical features, we observed that CTTAC and CTCCC haplotypes were associated with ER expression, and CTTAC haplotype showed a correlation with the status of PR. Therefore, these two haplotypes may provide valuable prognostic information for the survival of breast cancer patients.

## Conclusions

This study first established that SNPs in the ICOS gene may affect breast cancer risk and some SNPs were also associated with clinical characteristics of breast cancer in Chinese women from northeast of China. However, studies focusing on other critical polymorphisms in different populations and investigations of biological functions of ICOS gene polymorphisms remain to be further conducted.

## Abbreviations

CI: Confidence interval; ER: Estrogen receptor; HWE: Hardy-Weinberg equilibrium; *ICOS: Inducible costimulator; CTLA-4: Cytotoxic T lymphocyte antigen -4*; LN: Lymph node; OR: Odds ratio; PCR-RFLP: Polymerase chain reaction-restriction fragment length polymorphism; PR: Progesterone receptor; SNP: Single nucleotide polymorphisms; TZ: Tumor size.

## Competing interests

The authors declare that they have no competing interests.

## Authors' contributions

FYX designed the primers and wrote the drafts. DLL collected patients and controls blood samples. QJZ, ZKF, WGY and SC performed the PCR-RFLP experiments. JZ contributed to statistical analysis. DP and DJL conceived of the study, and participated in its design and coordination and helped draft the manuscript. All authors read and approved the final manuscript.

## Pre-publication history

The pre-publication history for this paper can be accessed here:

http://www.biomedcentral.com/1471-2407/11/392/prepub

## Supplementary Material

Additional file 1 **Figure S1**. Polymerase chain reaction-restriction fragment length polymorphism analysis of ICOS polymorphisms. This figure showed the restriction fragment length of each SNP (A for rs11889031, B for rs10932029, C for rs4675374, D for rs10183087 and E for rs10932037). Click here for file

Additional file 2 **Table S1**. ICOS polymorphisms and clinical features in cases in the study cohort and validation cohort. This table showed the positive results about the association between two SNPs (rs10932029 and rs11889031) and clinicopathologic features, including lymph node metastasis and the statuses of progesterone receptor (PR) and C-erbB-2. Click here for file
