# Supplementary material for: ICOS gene polymorphisms are associated with sporadic breast cancer: a case-control study
Source: BMC Cancer. 2011 Sep 15;11:392. doi: 10.1186/1471-2407-11-392 (PMC3185281; doi:10.1186/1471-2407-11-392)
Supplement: Additional file 1 — Figure S1. Polymerase chain reaction-restriction fragment length polymorphism analysis of ICOS polymorphisms. This figure showed the restriction fragment length of each SNP (A for rs11889031, B for rs10932029, C for rs4675374, D for rs10183087 and E for rs10932037). [file 1471-2407-11-392-S1.DOC]

**Figure S1.**


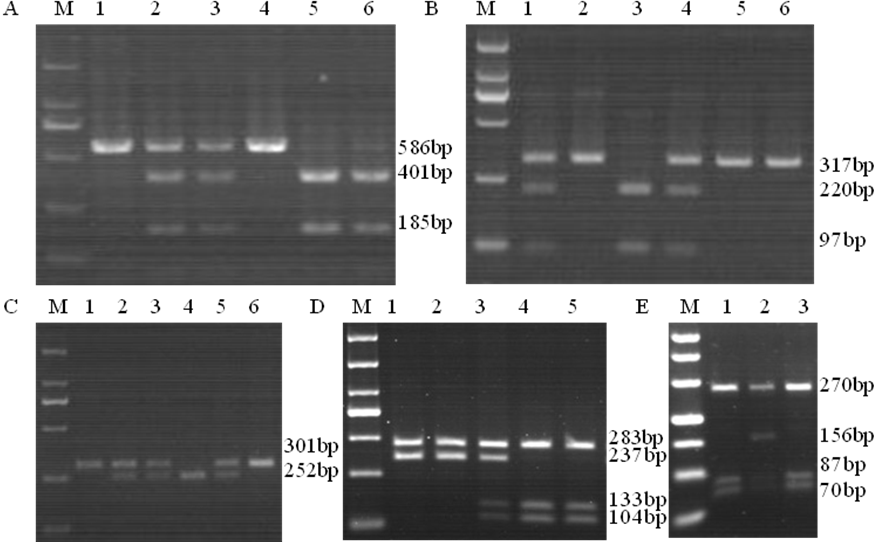


**Figure S1. Polymerase chain reaction–restriction fragment length polymorphism analysis of ICOS polymorphisms.** (A) Rs11889031 SNP: lanes 1 and 4: homozygote CC, lanes 2 and 3: heterozygote CT, lanes 5 and 6: homozygote TT. (B) Rs10932029 SNP: lanes 2, 5 and 6: homozygote TT, lanes 1 and 4: heterozygote CT, lane 3: homozygote CC. (C) Rs4675374 SNP: lanes 1 and 6: homozygote TT, lanes 2, 3 and 5: heterozygote CT, lane 4: homozygote CC. (D) Rs10183087 SNP: lanes 1 and 2: homozygote AA, lane 3: heterozygote AC, lanes 4 and 5: homozygote CC. (E) Rs10932037 SNP: lanes 1 and 3: homozygote CC, lane 2: heterozygote CT. M: Takara DNA Marker DL 2000 for A, B, and C, Takara DNA Marker DL 1000 for D, and Takara DNA Marker DL 500 for E.
