# Supplementary material for: ICOS gene polymorphisms are associated with sporadic breast cancer: a case-control study
Source: BMC Cancer. 2011 Sep 15;11:392. doi: 10.1186/1471-2407-11-392 (PMC3185281; doi:10.1186/1471-2407-11-392)
Supplement: Additional file 2 — Table S1. ICOS polymorphisms and clinical features in cases in the study cohort and validation cohort. This table showed the positive results about the association between two SNPs (rs10932029 and rs11889031) and clinicopathologic features, including lymph node metastasis and the statuses of progesterone receptor (PR) and C-erbB-2. [file 1471-2407-11-392-S2.DOC]

Table S1. *ICOS* polymorphisms and clinical features in cases in the study cohort and validation cohort

| Clinical features | SNP | Genotype and allele | Study cohort | | | | Validation cohort | | | |
| --- | --- | --- | --- | --- | --- | --- | --- | --- | --- | --- |
|  |  |  | Positive (%) | Negative (%) | OR (95% CI) | P value | Positive (%) | Negative (%) | OR (95% CI) | P value |
| C-erbB-2 | rs10932029 | TT | 178(89.45) | 266(83.91) | reference |  | 161(86.10) | 319(84.17) | reference |  |
|  |  | CT | 19(9.55) | 50(15.77) | 0.568(0.324,0.995) | 0.046 | 26(13.90) | 58(15.30) | 0.888(0.539,1.464) | 0.642 |
|  |  | CC | 2(1.01) | 1 (0.32) | 2.989(0.269,33.209) | 0.568 | 0(0) | 2(0.53) | — | 0.554 |
|  |  | T | 375(94.22) | 582(91.80) | reference |  | 348(93.05) | 696(91.82) | reference |  |
|  |  | C | 23(5.78) | 52(8.20) | 0.686(0.413,1.141) | 0.144 | 26(6.95) | 62(8.18) | 0.839(0.521,1.350) | 0.468 |
| PR | rs11889031 | CC | 171(44.88) | 38(29.23) | reference |  | 153(44.35) | 73(32.74) | reference |  |
|  |  | CT | 168(44.09) | 76(58.46) | 0.491(0.315,0766) | 0.002 | 154(44.64) | 122(54.71) | 0.602(0.418,0.869) | 0.006 |
|  |  | TT | 42(11.02) | 16 (12.31) | 0.583(0.297,1.145) | 0.115 | 38(11.01) | 28(12.56) | 0.648(0.369,1.136) | 0.128 |
|  |  | C | 510(66.93) | 154(58.78) | reference |  | 460(66.67) | 268(60.09) | reference |  |
|  |  | T | 252(33.07) | 108(41.22) | 0.705(0.528,0940) | 0.017 | 230(33.33) | 178(39. 91) | 0.753(0.588,0.964) | 0.024 |
| Lymph node  involvement |  | CC | 122(46.74) | 121(36.34) | reference |  | 128(46.04) | 115(34.53) | reference |  |
|  |  | CT | 110(42.15) | 171(51.35) | 0.638(0.451,0.903) | 0.011 | 120(43.17) | 181(54.35) | 0.596(0.423,0.838) | 0.003 |
|  |  | TT | 29(11.11) | 41(12.31) | 0.702(0.528,0.940) | 0.195 | 30(10.79) | 37(11.11) | 0.728(0.423,1.254) | 0.252 |
|  |  | C | 354(67.82) | 413(58.93) | reference |  | 376(67.63) | 411(61.71) | reference |  |
|  |  | T | 168(32.18) | 253(41.07) | 0.775(0.609,0.986) | 0.038 | 180(32.37) | 255(38.29) | 0.772(0.609,0.978) | 0.032 |

Abbreviations: PR=progesterone receptor; OR= odds ratio; CI = confidence interval
